# Early Real-World Experience with CoreValve Evolut PRO and R Systems for Transcatheter Aortic Valve Replacement

**DOI:** 10.1155/2019/1906814

**Published:** 2019-10-01

**Authors:** Gaurav Rao, Shikha Sheth, Joseph Donnelly, Andrew Scatola, Umair Tariq, Saaron Laighold, Cindy Grines, Bruce Rutkin

**Affiliations:** ^1^Department of Cardiology, North Shore University Hospital, Donald and Barbara Zucker School of Medicine at Hofstra/Northwell, Manhasset, NY, USA; ^2^Department of Internal Medicine, North Shore University Hospital, Donald and Barbara Zucker School of Medicine at Hofstra/Northwell, Manhasset, NY, USA; ^3^Department of Cardiovascular and Thoracic Surgery, North Shore University Hospital, Donald and Barbara Zucker School of Medicine at Hofstra/Northwell, Manhasset, NY, USA

## Abstract

**Objectives:**

The purpose of this study was to compare the efficacy and safety of the Evolut PRO to the Evolut R valve in a real-world setting.

**Background:**

The next-generation self-expanding transcatheter aortic valve replacement (TAVR) system, the CoreValve Evolut PRO was designed with an outer pericardial skirt to improve valve-sealing performance. Safety and efficacy of this valve have not previously been compared to its predecessor, the Evolut R valve.

**Methods:**

We retrospectively studied 134 patients who underwent TAVR with the Evolut PRO or Evolut R valve over one year at a tertiary center. Endpoints, defined by the Valve Academic Research Consortium-2 criteria, included device success, paravalvular leak (PVL), and a composite safety endpoint including mortality, stroke, major vascular complications, life-threatening bleeding, acute kidney injury, coronary artery obstruction, and repeat procedure for valve-related dysfunction.

**Results:**

60 Evolut PRO and 56 Evolut R patients met the study criteria. Both groups had similar device success rates (90 vs. 89%, *p*=0.44). Incidence of moderate PVL was similar on discharge (5 vs. 11%, *p*=0.68) and at 30 days (11 vs. 13%, *p*=0.79), with nil incidence of severe PVL. There were no mortalities, and the VARC-2 safety endpoint at 30 days was comparable.

**Conclusion:**

Despite the additional pericardial skirt and larger sheath size of Evolut PRO, outcomes were comparable between the two Evolut systems, supporting adoption of the newest generation valve in the management of severe aortic stenosis as well as continued use of the Evolut R in patients with smaller vasculature warranting a lower profile device.

## 1. Introduction

Transcatheter aortic valve replacement (TAVR) has become a treatment option for selected patients with symptomatic severe aortic stenosis [1]. Despite improved survival and superior hemodynamics compared to surgical aortic valve replacement (SAVR), [2–4] randomized, controlled trials demonstrated that patients undergoing TAVR consistently have higher rates of PVL as compared to SAVR, [4, 5] resulting in higher risk of adverse events and mortality [6, 7]. Calcium burden as well as the location of calcium in the aortic annulus and the aortic wall of each valve cusps has been implicated in predicting paravalvular leak (PVL) due to a potentially suboptimal seal [8, 9].

These limitations have led to iterative technological advancements in transcatheter heart valve (THV) design, with efforts to create lower profile, repositionable devices with optimal radial force and annular sealing. One notable innovation is the Evolut R self-expanding valve (Medtronic), the successor to the CoreValve prosthesis. The Evolut R valve comprises a shortened nitinol frame to optimize anatomical fit with a longer inner porcine pericardial sealing skirt. Two recent prospective trials involving this prosthesis demonstrated improved rates of moderate or severe PVL of 5.7% (at 30 days) and 1.9% (at discharge) and decreased need of new pacemaker implantation of 16.4% (at 30 days) and 19.3% (at discharge), respectively [10, 11].

The Evolut PRO transcatheter aortic valve represents the next generation of self-expanding valves in the CoreValve Evolut line. Using the Evolut R as a platform, the major design modification is an external porcine pericardial wrap added to cover the first 1.5 inflow cells with the intent to reduce PVL through an enhanced outer seal. The purpose of this study was to evaluate the efficacy and safety of the Evolut PRO system for the treatment of symptomatic severe aortic stenosis in a real-world, tertiary level center as compared to its predecessor, the Evolut R.

## 2. Methods

### 2.1. Patient Population

Inclusion criteria were the presence of severe symptomatic aortic stenosis in patients who were deemed high risk for SAVR (surgical aortic valve replacement) by a Heart Team, which included a minimum of one interventionist cardiologist and two cardiothoracic surgeons. Severe aortic stenosis diagnosis required an orifice area <1 cm^2^ by continuity equation and a mean aortic valve gradient >40 mm Hg or a maximal velocity >4.0 m/s at rest on transthoracic echocardiography. Multidetected computer tomography was used for anatomic assessments in all patients for aortic sizing as well as peripheral vascular suitability. Patients were assessed to be symptomatic if they had a NYHA functional class 2 or greater. Key patient exclusion criteria were any previous aortic valve implantation, bicuspid aortic valves, and patients requiring subclavian access.

All patients gave informed written consent after discussion of the risks and alternatives of TAVR. This study was conducted retrospectively, and the decision of the type of valve used for treatment was not affected by this study. Protocol for data collection was approved by the hospital Internal Review Board (IRB).

### 2.2. Procedural Details

The Evolut PRO valve was delivered via a 16 Fr equivalent catheter while the Evolut R valve was delivered via a 14 Fr equivalent catheter. An in-line sheath or a separate 20 Fr introducer sheath for Evolut PRO and an 18 Fr for Evolut R was used at the operators' discretion. The native valve was predilated with a balloon prior to valve delivery at the discretion of the operators. Once in position, both the Evolut R and PRO valves were deployed in stepwise fashion including repositioning as needed. The majority of the Evolut R valves and Evolut PRO valves were implanted under general anesthesia. The mean time to discharge after TAVR was approximately 6 days. All patients received aspirin (81 mg) and clopidogrel (75 mg) for at least 1 month, up to 12 months, or oral anticoagulants (OACs) as indicated. Patients with atrial fibrillation were restarted on their prior OAC regimen.

#### 2.2.1. Echocardiographic Analysis

All patients underwent transthoracic echocardiograms at baseline, before hospital discharge, and at 30 days. Postprocedural regurgitation was accessed using multiple parameters including regurgitation jet density and width and circumferential extent of turbulent regurgitation color jet around the aortic annulus for PVL. Additional parameters included descending and abdominal aorta diastolic flow reversal on pulsed wave Doppler and pressure one-half time of the aortic regurgitation on the continuous wave Doppler signal. Color flow imaging was given priority with trace or mild regurgitation associated with <10% circumferential AR extent, moderate (10–20%), and severe regurgitation with circumferential extent >20%. PVL grading was verified by two cardiologists.

#### 2.2.2. Endpoints

Primary endpoints for this study were the presence of paravalvular leak at discharge and 30 days after discharge and device success as defined by Valve Academic Research Consortium (VARC)-2 criteria [12]. Postprocedure device success at 1 to 7 days was assessed by composite of absence of procedural mortality, correct positioning of the valve as well as successful performance of the heart valve with postprocedural mean aortic valve gradient <20 mm Hg or peak velocity <3 m/s, and absence of moderate or severe prosthetic valve regurgitation. Secondary endpoints included the VARC-2 defined composite safety endpoints including mortality, stroke, major vascular complications, life-threatening bleeding, stage 2 or 3 acute kidney injury, coronary artery obstruction, major vascular complication, and valve-related dysfunction requiring repeat procedure. Additional clinical endpoints included were the development of a new left bundle branch block (LBBB) or the need for a new permanent pacemaker implantation.

### 2.3. Statistical Analysis

All statistical analyses were performed with SPSS 23. Student's *t*-test was used for continuous variables, including for comparison of baseline characteristics, demographics, and outcomes if the data were normally distributed; otherwise, the Mann–Whitney test for independent samples was used for comparison. The chi-squared test was used for categorical variables, and the Fischer test was used when appropriate. All comparisons were measured with a 2-sided alpha level of 0.05.

## 3. Results

Between January 2017 and November 2017, 134 patients with symptomatic severe aortic stenosis were implanted with either the Evolut R or Evolut PRO valve of which 116 were included in this study. 63 patients were treated with either 23 mm, 26 mm, or 29 mm Evolut Pro valve through November 2017 of which 3 were excluded for requiring subclavian access. The 60 patients were compared with the patients undergoing treatment with Evolut R valves between January and November 2017. There were 71 patients treated with 23 mm, 26 mm, 29 mm, or 34 mm Evolut R valves, of which 12 patients undergoing TAVR in the setting of a prior SAVR were excluded as were 3 patients requiring subclavian access.

The patient population had a mean age of 84.6 in the Evolut PRO group and 80.4 in the Evolut R group with a Society of Thoracic Surgeons (STS) risk score of 7.1% and 6.5%, respectively. Comorbidities were similarly distributed between both groups with the exception of a statistically significant older age distribution in the Evolut PRO group (*p*=0.02) and gender difference between the groups (18% men in PRO vs. 46% in R, *p*=0.001), both of which were unintentional. Baseline characteristics are listed in Table 1. 21.7% of the patients in the PRO group and 14.3% in the R group had a pre-existing permanent pacemaker.

### 3.1. Procedural Outcomes

Of the 116 patients receiving Evolut valve implants, 60 received PRO and 56 received R. Procedural outcomes are summarized in Table 2. All but three valves in each group were implanted via transfemoral access; the others were via subclavian access which were excluded from analysis. All patients included in the study had valve implantation via iliofemoral access. In the Evolut PRO group versus R group, respectively, 3 patients (5%) versus 2 patients (3.5%) received a 23 mm valve, 28 patients (46.7%) versus 18 (27.3%) received a 26 mm valve, and 29 patients (48.3%) versus 19 (33.9%) received a 29 mm valve. Additionally, 17 patients (30.4%) received a 34 mm valve in the Evolut R group, which is a size not available as Evolut PRO. There was a statistically significant difference in mean valve size between the two groups (*p*=0.004). Pre-TAVR balloon aortic valvuloplasty was performed in 11 (18.3%) patients in the PRO versus 14 (25%) patients in the R group (*p*=0.5). Post-dilation was performed in 16 PRO patients (26.7%), of whom 6 also had predilation, and 15 R patients (26.8%), 3 of whom had predilation (*p*=0.8). Rates of repositioning for Evolut PRO and R during deployment were not available for analysis.

### 3.2. Device Success

Both Evolut PRO and Evolut R groups had similar device success rates, 90% and 89.3%, respectively (*p*=0.44). Three patients in the Evolut PRO group and 1 patient in the Evolut R group had a mean transvalvular gradient (≥20 mm Hg). Three other patients in the Evolut PRO group had moderate PVL, while 6 patients in the Evolut R group, including the 1 with an elevated gradient, had moderate PVL. Breakdown of individual points is listed in Tables 3 and 4.

### 3.3. Clinical Outcomes

**Figure 1:**
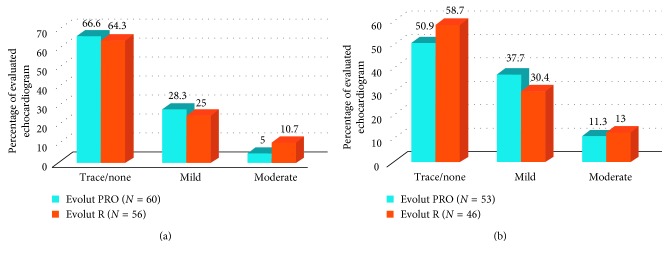
Paravalvular leak at discharge and 30 days. Legend: Echocardiographic findings of paravalvular leak (PVL) at discharge (a) and 30 days (b) for patients implanted with CoreValve Evolut PRO or CoreValve Evolut R. There is no significant difference between the two groups.

The rate of none or trace PVL was similar between the two groups, (66.6% Evolut PRO vs. 64.3% Evolut R, *p*=0.65). There was no difference in the incidence of moderate PVL at discharge (5.0% PRO versus 10.7% R, *p*=0.25) (Table 4). There was no incidence of severe PVL in any patients. At 30 days, echocardiograms were available for 53 of the Evolut PRO and 46 of the Evolut R patients. The rate of none or trace PVL remained similar though decreased from discharge (50.9% Evolut PRO vs. 58.7% Evolut R, *p*=0.73). There was no significant difference in moderate PVL between the two groups at 30 days (11.3% PRO versus 13% R, *p*=0.79) (Figure 1).

Two patients in the Evolut PRO group and one in the Evolut R group had disabling strokes. There were no mortalities or MIs in either group at 30 days. Major vascular complications occurred in 3 patients in both groups, all relating to access sites. Three patients experienced life-threatening or major bleeding in the PRO group while 2 patients experienced a major bleed in the R group. Four patients in the Evolut PRO group and 3 in the Evolut R group met the VARC-2 composite endpoint criteria, with similar safety demonstration in both groups at 30 days (93.3% Evolut PRO and 94.6% Evolut R, *p*=0.76) (Table 5). In a subanalysis of the composite endpoints of death, MI, and stroke, rates were similar between Evolut PRO and Evolut R (96.7% versus 98.2%, respectively, *p*=0.60). Safety profile is similar with respect to bleeding alone, 95% with Evolut PRO and 96.4% with Evolut R (*p*=0.71).

### 3.4. Additional Endpoints

**Figure 2:**
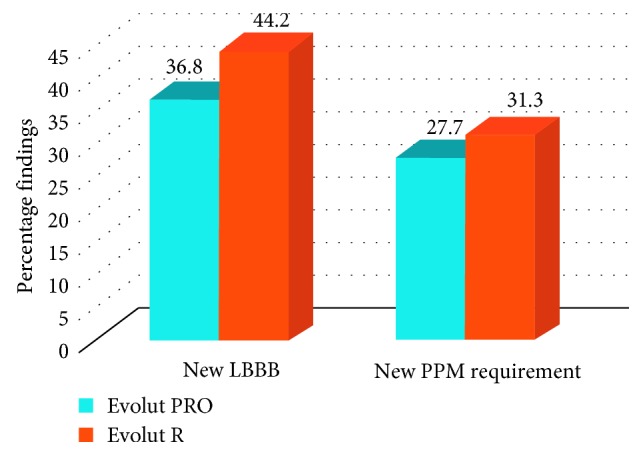
New left bundle branch block and pacemaker requirement. Legend: New electrocardiogram findings of left bundle branch block (LBBB) and new permanent percutaneous pacemaker (PPM) requirement in the patients implanted with CoreValve Evolut PRO and CoreValve Evolut R. There is no significant difference between the two groups.

A new LBBB was diagnosed in 21 patients in the Evolut PRO group (36.8%) and 23 patients in the Evolut R group (44.2%) prior to discharge (*p*=0.43). Additionally, 13 patients in the Evolut Pro (27.7%) and 15 patients in the Evolut R group (31.3%) required new implantation of a permanent percutaneous pacemaker (PPM) (*p*=0.70). There was no significant difference in rates of new LBBB or PPM implantation between the two groups (Figure 2). Patients who had a LBBB (3 patients in the PRO group and 4 patients in the R group) or PPM (13 patients in the PRO group and 8 patients in the R group) prior to valve implantation were excluded from this analysis.

## 4. Discussion

TAVR has evolved to be an acceptable treatment choice for patients with severe symptomatic aortic stenosis across a spectrum of surgical risk [4, 13]. The widespread adoption of the procedure has been driven by increased experience as well as successive technological advancements. We report the clinical outcomes of the first 60 patients who underwent TAVR with the new generation Evolut PRO valve at our tertiary center and compared them to the outcomes with the prior generation Evolut R valve implanted during the same time period.

### 4.1. Paravalvular Leak

Post-TAVR PVL has been known to be an independent predictor of increased late mortality [14, 15]. In our study, we found that 5% of the patients with Evolut PRO implant had moderate PVL at discharge compared to 10.7% receiving Evolut R, without any occurrence of severe PVL in either group. The incidence of moderate PVL increased at 30 days more so in the Evolut PRO cohort comparatively (11.3% moderate PVL in the Evolut PRO versus 13% in the Evolut R group), making rates similar between both groups. Our results suggest that the durability of the porcine pericardial wrap of the Evolut PRO did not confer benefit at 30 days as compared to on discharge as compared to the Evolut R. The cause of this is unclear but this signal requires further investigation. Operator dependency may have had an effect on this finding given the early experience. Additionally, our rates are higher compared to the CoreValve Evolut R US trial finding 5.3% moderate/severe PVL at 30 days [2] and the recent CoreValve Evolut PRO clinical trial finding 0% [16]. We noticed decreasing rates of PVL over the time course, suggesting operator comfort with repositioning and lower valve implantation likely, played a role. Our results demonstrated excellent device success rates and safety profiles comparable between the two groups and superior to the first generation of CoreValve, which had a device success rate of 78.6% [17]. Importantly, mortality remained zero in our study, the stroke rate was 1.8%, and there was no periprocedural or postprocedural myocardial infarction, which were similar outcomes as prior reported trials [16, 18].

The Evolut PRO has an external pericardial wrap designed to reduce prosthetic valve regurgitation. The Evolut R improved upon the CoreValve design by reducing the height of the valve, while preserving the inner pericardial skirt and allowing more accurate repositioning during deployment. The Evolut PRO has an added outer pericardial wrap that potentially offers a more secure seal to diminish PVL. Its design improves upon the Evolut R design at the expense of a slightly larger minimum vessel diameter (5.5 mm for PRO vs. 5 mm for R) and introducer sheath size (16 Fr vs. 14 Fr) requirement with current availability in three valve sizes (23 mm, 26 mm, and 29 mm). Our study demonstrated that despite the increase in sheath size, the safety profile of the Evolut PRO valve remains comparable to the Evolut R with low rates of vascular complications and bleeding events.

Two notable findings in our study were an unintentionally higher age and skew towards female patient selection for the patients receiving Evolut PRO valves. As this study is retrospective analysis, the selection of patients for each arm was operator dependent. This difference in selection could potentially be due to smaller valve size availability in Evolut PRO selection that is not offset by the increase in requirement of minimal vessel diameter. As our study demonstrates comparable safety and efficacy of the two devices, a larger, blinded study is warranted to further investigate possible gender differences between the two groups.

### 4.2. Pacemakers

Anatomically, the left bundle runs along the noncoronary cusp before traversing through the membranous septum. The interaction of the prosthetic valve at the level of membranous septum has the potential to create conduction system disturbances. Presence of a pre-existing RBBB or LBBB, increased noncoronary cusp thickness, diameter of the membranous septum, and calcium burden and location in the left coronary cusp have all been implicated in predicting the risk of conduction disturbances requiring pacemaker implantation [19, 20]. Our study demonstrated similar rates of postprocedural PPM implantation between the Evolut PRO and R groups (27.7% and 31.3%, respectively), higher than reported in the prior Evolut PRO and R clinical trials (11.8% and 19.7%, respectively), however similar to that reported recently in the SOLVE-TAVI trial that was comparing SAPIEN 3 with Evolut R (22.9% and 19.0%, respectively) [10, 16, 21]. Many of our patients had pre-existing RBBB or LBBB placing them at a higher risk (16.6% PRO and 19.6% R groups). Though the initial concern for the Evolut PRO valve was related to the risk of interaction with the conduction system from the outer pericardial wrap, the reason for higher pacemaker implantation rate at our site is likely multifactorial. The self-expanding nature of the valve may have resulted in slow conduction damage over the first 1-2 days after procedure. A previous study showed that the SAPIEN 3 valve, which had an additional polyethylene terephthalate fabric seal around the inflow portion of the valve, resulted in increased incidence of pacemakers at 30 days compared to its predecessor [22]. Another prior comparison of the SAPIEN 3 valve to the Evolut R valve revealed a significantly higher incidence of pacemaker implantation in the Evolut R group [23]. Both the SAPIEN 3 and Evolut PRO valves have a similar outer wrap; however, one of the major differences between the two valves is that SAPIEN 3 is balloon expandable while the Evolut R is self-expandable.

### 4.3. Limitations

This study is limited by its retrospective, nonrandomized design at a single center which subjects it to the inherent weakness of such analysis. Specifically, unmeasured bias could have affected the choice of one valve or therapy over another. However, this analysis is valuable in comparison to the randomized trial by Medtronic as it is reflective of a real-world clinical practice in which valve choice decisions are made by a Heart Team and based on patient's baseline characteristics. Secondly, as addressed earlier, the limitation of the Evolut PRO design to a smaller annular range resulted in a skew of the patient population towards women of older age, specifically who were likely at higher STS risk. Thirdly, our study reflects an early experience as our follow-up was only 30 days. A longer follow-up will be necessary to make definitive considerations between the two valve types with regard to durability of device success and absence of PVL over time. However, in the short period, the similar safety and efficacy outcomes of both valves are favorable, especially in patients who are generally at higher risk for adverse events.

## 5. Conclusions

As compared to the Evolut R valve, the Evolut PRO valve is a reasonable alternative for TAVR in patients with an equivalent safety profile and hemodynamics in the early setting. In patients with smaller vascular access diameters, Evolut R would be a reasonable choice without compromising safety or efficacy. Larger, prospective studies are required to identify other subgroups who would benefit from the newer generation Evolut Pro valve.

## Figures and Tables

**Table 1:** Baseline characteristics.

	Evolut PRO (*N* = 60)	Evolut R (*N* = 56)	*p* value
Age (years)	84.6 ± 6.3	80.4 ± 9.4	0.02
Gender (M/F)	11/50	26/30	0.001
BMI	27.5 ± 6.8	28.8 ± 7.2	0.13
STS score	7.1 ± 4.0	6.5 ± 4.8	0.13
ESRD on HD	1	2	0.51
Hypertension	54	53	0.35
Peripheral artery disease	7	14	0.06
Diabetes mellitus	18	21	0.40
Chronic lung disease	17	23	0.15
Prior PCI	24	23	0.90
Prior CABG	9	16	0.08
Prior MI	15	10	0.35
Prior atrial fibrillation/atrial flutter	15	11	0.49
Prior PM	13	8	0.30
Prior LBBB	3	4	0.63
Prior RBBB	7	7	0.89

Values are mean ± standard deviation or *n*.

**Table 2:** Procedural outcomes.

	Evolut PRO (*N* = 60)	Evolut R (*N* = 56)	*p* value
General anesthesia	59 (98.3)	46 (82.1)	0.002
Implanted valve size, mm			
23	3 (5.0)	2 (3.5)	0.71
26	28 (46.7)	18 (27.3)	0.11
29	29 (48.3)	19 (33.9)	0.12
34	—	17 (30.4)	—
Preimplant balloon valvuloplasty	11 (18.3)	14 (25.0)	0.38
Postimplant balloon valvuloplasty	16 (26.7)	15 (26.8)	0.99
Length of stay, days	6.4 ± 5.6	6.6 ± 5.1	0.17

Values are *n* (%), mean ± standard deviation.

**Table 3:** Device success at discharge.

	Evolut PRO (*N* = 60)	Evolut R (*N* = 56)	*p* value
Absence of procedural mortality	60 (100)	56 (100)	1.00
Correct positioning of single valve in proper anatomical location	60 (100)	56 (100)	1.00
Intended performance of prosthetic heart valve			
No prosthesis-patient mismatch	60 (100)	56 (100)	1.00
Mean gradient <20 mm Hg or peak velocity <3 m/s	60 (100)	55 (98)	0.79
Absence of moderate or severe prosthetic regurgitation	57 (95)	50 (89)	0.25
Overall device success	54 (90.0)	50 (89.3)	0.90

Values are *n* (%).

**Table 4:** Discharge and 30-day echocardiogram findings.

	Evolut PRO	Evolut R	*p* value
PVL—discharge			
Trace/none	40/60 (66.6)	36/56 (64.3)	0.65
Mild	17/60 (28.3)	14/56 (25)	0.68
Moderate	3/60 (5)	6/56 (10.7)	0.25
Severe	0/60	0/56	—
Aortic valve function—discharge			
EF	67.9 ± 10.3	65.8 ± 9.4	0.19
Mean transaortic valve gradient (mm Hg)	8.5 ± 4.6	8.2 ± 4.0	0.99
Maximum aortic velocity (m/s)	1.9 ± 0.4	1.9 ± 0.4	0.78
Aortic valve area (cm^2^)	1.9 ± 0.4	1.9 ± 0.5	0.45
Aortic valve area index (cm^2^/m^2^)	1.4 ± 0.7	1.1 ± 0.3	0.007
PVL—30 days^*∗*^			
Trace/none	27/53 (50.9)	25/46 (58.7)	0.73
Mild	20/53 (37.7)	14/46 (30.4)	0.45
Moderate	6/53 (11.3)	6/46 (13.0)	0.79
Severe	0/53	0/46	—
Aortic valve function—30 days			
EF	62.2 ± 11.3	66.6 ± 9.3	0.62
Mean transaortic valve gradient (mm Hg)	7.0 ± 3.3	8.1 ± 4.3	0.19
Maximum aortic velocity (m/s)	1.8 ± 0.4	1.8 ± 0.5	0.69

Values are mean ± standard deviation, *n*/*N* (%). ^*∗*^Note that 30-day echocardiogram findings were not available for the entire population. Aortic valve area and aortic valve area index not available at 30 days.

**Table 5:** Clinical outcomes and safety endpoints.

	Evolut PRO (*N* = 60)	Evolut R (*N* = 56)	*p* value
Mortality			
Prior to discharge	0	0	—
30 days	0	0	—
Myocardial infarction	0	0	—
Stroke			
Ischemic	1 (1.7)	1 (1.8)	0.96
Hemorrhagic	1 (1.7)	0	0.33
Vascular complications			
Major	3 (5.0)	3 (5.4)	0.93
Minor	3 (5.0)	7 (12.5)	0.15
Percutaneous closure—device failure	0 (3.3)	0	0.17
Bleeding			
Life-threatening	2 (3.3)	0	0.17
Major	1 (1.7)	2 (3.6)	0.52
Acute kidney injury			
Stage 1	3 (5.0)	8 (14.3)	0.09
Stage 2	0	2 (3.6)	0.14
Stage 3	1 (1.7)	1 (1.8)	0.96
Embolization/migration	0	0	—
Endocarditis	0	0	—
Valve thrombosis	0	0	—
Coronary artery obstruction	0	0	—
Early safety at 30 days	56 (93.3)	53 (94.6)	0.76
New LBBB	21/57 (36.8)	23/52 (44.2)	0.43
New PPM requirement	13/47 (27.7)	15/48 (31.3)	0.70

Values are *n* or *n*/*N* (%). The group of patients with pre-existing LBBB or PPM was excluded for comparison.

## Data Availability

The data used to support the findings of this study are restricted only to the authors of this study by the Institutional Review Board at Northwell Health in order to protect patient privacy. The corresponding author will consider request for general access to these data after publication of this article; however, approval will be at the discretion of the Northwell Health Institutional Review Board.

## Abbreviations

LBBB: Left bundle branch block
OAC: Oral anticoagulation
PPM: Permanent percutaneous pacemaker
PVL: Paravalvular leak
RBBB: Right bundle branch block
SAVR: Surgical aortic valve replacement
STS: Society of Thoracic Surgeons
TAVR: Transcatheter aortic valve replacement
VARC: Valve Academic Research Consortium
THV: Transcatheter heart valve.
